# *Helicobacter pylori* Infection Mass Screening for Children and Adolescents: a Systematic Review of Observational Studies

**DOI:** 10.1007/s12029-021-00630-0

**Published:** 2021-03-24

**Authors:** Hiroaki Saito, Yoshitaka Nishikawa, Yuko Masuzawa, Masaharu Tsubokura, Yasuhiro Mizuno

**Affiliations:** 1 Department of Gastroenterology, Sendai Kousei Hospital, Sendai, Miyagi Japan; 2 Department of Radiation Health Management, Fukushima Medical University School of Medicine, Fukushima, Fukushima Japan; 3 Department of Health Informatics, Kyoto University School of Public Health, Kyoto, Kyoto Japan; 4 Chiba Faculty of Nursing, Tokyo Healthcare University, Funabashi, Chiba Japan; 5 Marru-Clinic Yokosuka, Yokosuka, Kanagawa Japan

**Keywords:** *Helicobacter pylori*, Mass screening, Adolescent, Child

## Abstract

**Purpose:**

Population-based *Helicobacter pylori* (*H. pylori*) screening and eradication for adults in areas with a high incidence of gastric cancer have been shown to be effective. The current status of *H. pylori* screening for young people, however, has not been sufficiently evaluated.

**Methods:**

A systematic review of population-based *H. pylori* screening of young people was performed using four databases (MEDLINE, EMBASE, the Cochrane Library, and ICHUSHI) and independently evaluated by two investigators. Studies were evaluated with regard to the country, region, screening method, target age, number of screened people, and rate of positive screening.

**Results:**

From 3231 studies, 39 studies were included (14 English original studies published in peer-review journals, 6 Japanese original studies, and 19 conference reports). These studies originated from 10 countries, with the largest number stemming from Japan (29 studies) followed by Germany (2 studies). Screening was performed using the urea breath test, blood antibodies, stool antigens, and urine antibodies. Five countries used the breath test as the first screening method, five used blood samples, two used stool antigens, and only Japan used urinary tests.

**Conclusion:**

Screening for *H. pylori* in young people was reviewed based on reports from several countries, and findings suggest that local authorities considering screening for *H. pylori* in young people need to scrutinize the age and potential methods. Further research is required to determine the effectiveness of mid- to long-term *H. pylori* screening for young people.

**Supplementary Information:**

The online version contains supplementary material available at 10.1007/s12029-021-00630-0.

## Background

Screening is performed to detect specific diseases among healthy people. There is a wide range of diseases that can be screened for, including infectious diseases [1], chronic diseases [2], and cancer [3, 4]. Screening enables the provision of early treatment to those with positive results and, from a public health perspective, also serves to prevent the spread of infectious disease such as tuberculosis [5], human immunodeficiency virus [6], and syphilis [7], in certain populations. In most cases, the parent organization providing the screening is a public organization, government, or local government. The aim of the screening programs is to prevent the prevalence of disease in a population and the emergence of disease in the future, as well as to enhance the well-being of the population and reduce the social burden of disease. Medical examinations are also conducted where companies provide opportunities for their employees to get tested. The type of screening these entities provide depends on the prevalence a specific disease in the population, cost-effectiveness of screening, and the economic status of the entity. For this reason, the structure and coverage of screening often vary widely across countries and regions. In particular, screening for cancer, which has a higher incidence and is the leading cause of death, is prioritized over communicable diseases, which have a lower mortality in developed countries. In recent years, however, several infectious diseases, including *Helicobacter pylori* (*H. pylori*), human papillomavirus, hepatitis virus B and C, and others have been shown to pose a risk for the development of cancer and chronic diseases, and this has become a public health challenge in both developing and developed countries [8–10].

*H. pylori* infection causes chronic gastritis and gastroduodenal ulcers and is a major risk factor for gastric cancer. In 1994, the International Cancer Research Agency recognized *H. pylori* as a class I carcinogenic pathogen to humans. [11–13] Eradication of *H. pylori* is an effective method for reducing the incidence of gastric cancer. Following endoscopic treatment for early-stage gastric cancer, eradication of *H. pylori* can have a significant inhibitory effect on gastric carcinogenesis [14]. Furthermore, eradication of *H. pylori* has also recently been shown to reduce the risk of gastric cancer in asymptomatic adults. [15] However, the effect of *H. pylori* eradication on the risk of gastric cancer has been shown to vary based on the region and race [16]. For example, studies have indicated that it is more effective to eradicate *H. pylori* in Asian populations with a high incidence of stomach cancer than in Americans [17]. The Asia–Pacific and European guidelines recommend screening for *H. pylori* in populations at high risk of gastric cancer, regardless of their symptoms. [18, 19] In Japan, multiple *H. pylori* screening and treatment programs are being conducted in the municipality for adults [20].

While there is a consensus that population-based *H. pylori* screening and eradication for adults in areas with high gastric cancer incidence is effective in reducing the incidence of gastric cancer, there is controversy regarding *H. pylori* infection therapy in young people. The benefit of *H. pylori* eradication in children and adolescents has not yet been clearly established. *H. pylori* is believed to be infectious in children [21]. It is thought that if *H. pylori* is eradicated at a young age, reinfection is less likely to occur and eradication in children with dyspepsia has been shown to be effective in improving symptoms [22]. However, whether the test and treat approach in young asymptomatic patients suppresses the long-term development of gastric cancer has yet to be established. Moreover, there is concern that treatment of asymptomatic *H. pylori*-infected patients may encourage the growth of drug-resistant *H. pylori*, which has already been a problem [23]. On the other hand, in order to prevent the carcinogenic effects of *H. pylori*, it is also believed that the earlier the eradication, the better. Pathologically, eradication of *H. pylori* prior to the progression of chronic gastritis and development of intestinal metaplasia is thought to be effective in controlling carcinogenesis, and therefore, the theory of eradication therapy in young people has merit [24, 25]. For this reason, several municipalities in Japan are offering *H. pylori* screening for teenagers [26]. Although there has been no clear consensus on the optimal timing of *H. pylori* eradication, the appropriate eradication strategy for young patients with *H. pylori* infection needs to be thoroughly discussed.

Although the pros and cons of *H. pylori* testing and eradication for young people need further and continued analysis, providers of screening programs need to consider its priority. However, despite the clinical need, there have been few reports on the implementation of *H. pylori* screening in young people. In the present report, we conducted a systematic review to determine whether there is a systematic trend in the implementation of *H. pylori* screening in Japan and around the world with the purpose of assessing the current status of *H. pylori* screening for young people.

## Methods

In this study we applied the guidelines for conducting a review from Parts 1 and 2 of the Cochrane Handbook for Systematic Reviews of Interventions version 6 and adhered to the systematic reporting guidelines of the preferred reporting items for systematic reviews and meta-analysis (PRISMA) statement. The PRISMA checklist is shown in Supplementary file 1. Our review protocol is shown in Supplementary file 2.

### Criteria for Considering Studies for This Review

#### Types of Studies

In this review, we included available published or unpublished observational studies and conference reports assessing the *H. pylori* infection. Reviews, survey reports for the purpose of infection rate identification, and case reports that included less than 10 cases were excluded.

#### Types of Participants

Healthy, asymptomatic persons, including teenagers, screened for *H. pylori* (with towns or schools being the smallest population units) were included in this review. Patients with symptoms were excluded.

### Search Methods for Identification of Studies

We searched MEDLINE (via PubMed, 1966 to 10 December 2018), EMBASE (1966 to 10 December 2018), Cochrane Library (Issue 12 of 12, December 2018), and ICHUSHI (1970 to 10 December 2018), for studies published in either English or Japanese using the search terms “*H. pylori* infection” and “child.” The search strategy is detailed in Supplementary file 3.

We also performed a search for additional references, by hand, from The Japanese Journal of Helicobacter Research and The Journal of Japanese Gastroenterological Association.

### Data collection and Analysis

#### Selection of Studies

Two authors (HS, YN) independently assessed all potential studies identified using our search strategy for inclusion in this review. Any disagreement was resolved through discussion or, if necessary, by consultation with a third author (YM).

#### Data extraction and Management

HS extracted the data using a standardized form. The items on the form included study design, countries, cities, participants, methods used for testing (e.g., urine, blood, urea breath), and positive rate.

#### Assessment of Risk of Bias in Included Studies

Due to this review including observational studies that utilized a variety of methods, we did not evaluate risk of bias.

#### Data Synthesis

Descriptive summaries of the individual study results are provided for each outcome in table including the characteristics of each review by country and by region within Japan.

## Results

### Description of Included Reviews

**Fig. 1:**
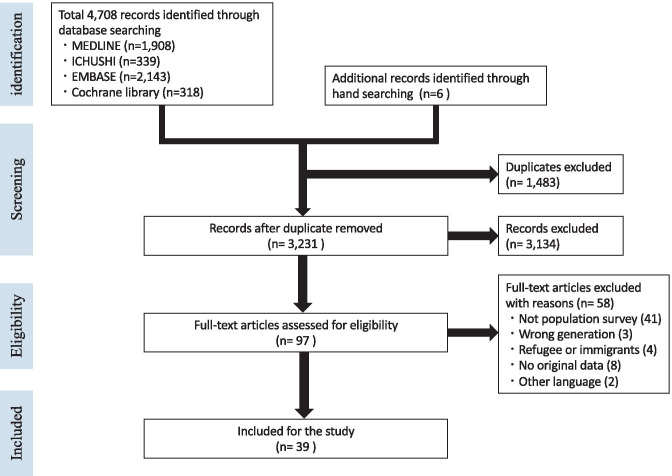
Flow diagram

The flow diagram of the selection process is shown in Fig. 1. A total of 4708 studies were identified from the database search. After removing duplicates, 3231 studies remained. A total of 3133 titles and abstracts were excluded as they did not involve human subjects, did not include young people, or the full text was not available in English. Of the 97 studies remaining, 58 were excluded because they had no illiquidity of testing to specific populations, such as sampling for research purposes and reporting only those that visited the hospital, and three reports were excluded as they were intended for refugees. The excluded studies are detailed in Supplementary file 4. Finally, 39 studies met the inclusion criteria for analysis of which 14 were English original studies published by peer-review journals [26–39], 7 Japanese original studies, [40–46], and 18 conference reports. [47–64]

### Study Characteristics

The 39 studies were reported from 10 countries, with the largest number stemming from Japan (29 studies) followed by Germany (2 studies). Screening was performed using the urea breath test, blood antibodies, stool antigens, and urine antibodies; five countries used the urea breath test as their first method of screening, five used blood samples, two used stool antigens, and only Japan used urinary tests. The age range of the subjects varied from country to country and from report to report (Table 1). The size of the population tested for *H. pylori* also varied between studies, ranging from school-based surveys to town or city-wide populations. Furthermore, the reported positive rates significantly differed between studies with the reported rates ranging from 2.6 to 72.4%. In Japan, four original articles in English peer-reviewed journals, seven original articles in Japanese journals, and 18 conference abstracts were published. Eleven prefectures and 17 cities or towns conducted screening for *H. pylori* in young people. Characteristics of the prefecture screenings are shown in Table 2. The most common test used for primary screening was the urine antibody test conducted in nine prefectures, followed by blood antibodies in three prefectures, and the fecal antigen test and urea breath test in two prefectures. The reported positive rate of *H. pylori* was low in Japan (2.6%-9.5%) compared with that in other countries. The main stakeholders providing screening were local governments in prefectures or municipalities, local medical associations, and university hospitals; in some areas, these branches worked together to deliver *H. pylori* screening programs.

**Table 1:** The countries and area reporting *H. pylori* screening for child and adolescent

Country	Area	Year	Age (yr)	Screening method^§^	Number screening participants	Positive rate
JAPAN [26, 37–64]	Various*	2007–2017	0–18	Urine, breath, stool, blood	62–3251	2.6–9.5%
Poland [27]	Grudziadz	2008–2015	13~17	Breath	3067	23.6%
South Korea [28]	-	2007	16≦	Blood	15,916	56.0%^a^
Uganda [29]	Kampala	2010^b^	0~12	Stool	427	44.3%
Egypt [30]	Cairo, Giza or Sohag	2007^b^	6~15	Breath	286	72.4%
Brazil [31]	Salvador	2005	4~11	Blood	1104	28.7%
China [32]	-	2008^b^	6~19	Breath	2480	13.1%
Finland [33]	Vammala	1996–2000	15	Blood	716–3326^*^	3.2–12.2%
Germany [34, 35]	Ulm, Erbach, Ehingen	1996–1998	5~8, 12–16	Breath	863–1143	11.3–13.7%
Italy [36]	Campogalliano	1999^b^	12~65	Blood	3289	59.7%^a^

^*^Listing in Table 2

^a^Including the positive rate of *H. pylori* in both adolescents and adults

^b^The year of publication of the paper as the research years were not included in the paper

^c^Urine for urine antibodies, blood for blood antibodies, breath for urea breath test, and stool for stool antigen test, respectively

**Table 2:** The lists of the prefectures in Japan reporting *H. pylori* screening for child and adolescent

Prefecture (city, town)	Year	Age (yr)	Screening method^†^	Number screening participants	Positive rate
Tottori (Hokuei town) [47]	2015	14~15	Urine	123	7.3%
Osaka (Takatsuki city, Nagaoka city) [48, 49]	2014, 2018^b^	13~14	Urine	1764,2173	6.6%, 3.9%
Hyogo (Sasayama city) [40, 41, 50–54]	2007–2016	12~17	Urine, blood	335–3251	3.1–5.8%
Hokkaido (Wakkanai city, Sapporo city, Abashiri city, Kinobetsu town, Bihoro town, Memuro town) [42–44, 55, 56]	2010–2016	0.5~18	Breath, urine, stool	795–836	4.5–8.9%
Saga* [57]	2017^b^	14~15	Urine	-	4.2%
Okayama (Maniwa city) [58]	2013	12~15	Urine, breath	317	4.4%
Nagano (Matsumoto city) [26, 37, 38, 46, 59, 60, 64]	2007–2015	12~17	Blood, urinary	126–3251	2.6–9.5%
Yamagata (Murayama city) [61]	2016^b^	13~14	Blood	233	5.6%
Kyoto [45]	2015–2017	15~16	Urine	1955	6.6%
Gihu (Higashi Shirakawa city) [62]	2015	13~15	Stool	62	4.8%
Akita (Yurihonjo city, Nikaho city) [39, 63]	2015–2016	13~15	Urine	1765	5.4%

^*^All junior-high school students aged 14–15-year-old in the prefecture were eligible for the screening

^a^Urine for urine antibodies, blood for blood antibodies, breath for urea breath test, and stool for stool antigen test, respectively

^b^The year of publication of the paper because the year of research was not mentioned in the paper

## Discussion

### Summary

In this study, we clarified, for the first time, the present status of *H. pylori* screening for young people worldwide. We screened 3231 reports, of which 97 reports were reviewed and 39 were included in our analysis. We then summarized the results, by country, in terms of screening methods, number of screenings, and positive rates. This review was independently screened by two reviewers, aiming to reduce selection bias. However, it should be noted that we only included English and Japanese literature; furthermore, we included grey literature and there were limitations with regards to assessing the quality of the literature.

### Demographic of the *H. pylori* Screening for Young People

The prevalence of gastric cancer varies greatly by country and region, and differences in regional *H. pylori* infection rates and *H. pylori* pathogenicity have an impact on said prevalence [65–67]. For this reason, it is thought that strategies for *H. pylori* treatment for the prevention of gastric cancer will differ between populations. Currently, screening for *H. pylori* is recommended in areas with high *H. pylori* infection rates and a high incidence of gastric cancer. [18, 19] Our study, however, included countries from areas with both high and low incidences of gastric cancer and noted substantial differences in the age-standardized incidence rate (ASR) between countries. The ASR of gastric cancer per 100,000 of population in the countries included in this study are reported to be 39.6 in South Korea, 27.5 in Japan, 20.7 in China, 8.3 in Poland, 7.9 in Brazil, 7.2 in Italy, 6.7 in Germany, 4.2 in Finland, 3.9 in Uganda, and 2.8 in Egypt. Eradication of *H. pylori* is a preventive measure against gastric cancer, as well as a preventive measure against peptic ulcers, MALT lymphoma, iron deficiency anemia, and immune thrombocytopenic purpura, issues that can arise in children and young people [68–70]. It is possible that efforts to screen young people for *H. pylori* in areas with a low incidence of gastric cancer may not be aimed solely at the preventive effect of gastric cancer.

The age at which the screening approach should be initiated is controversial. This review indicated that a proportion of the studies included, especially those from Japan, were found to be screening young people at junior high school. This is because junior high school education is compulsory in Japan, an environment that allows for the targeting of all young people in the region. Even if screening is done at an early age, such as elementary school, therapeutic intervention then raises concerns with regard to antibiotic capacity and side effects. As such, it would be potentially be more effective to introduce screening as part of a junior high or high school health check-up. It is theorized that these “test and treatment” efforts will contribute to reducing the incidence of gastric cancer in the population and will also contribute to reducing the prevalence of *H. pylori* in the future. However, long-term trends require ongoing monitoring.

### Screening Method

The screening method reported in the study included a urine test, blood test, stool test, and urea breath test. Of these, urine tests were used only in Japan. Although this study did not include a detailed inspection, the sensitivity and specificity of the test, the cost of the test, and the simplicity of the test were the main factors in the selection of the testing method for screening. The sensitivity and specificity of the fecal antigen test and urea breath test are higher than those of blood and urine antibodies test. The cost is generally higher for the urea breath test, which requires reagents. Urea breath tests and urine tests are not invasive, but blood antibodies require a blood draw. Some believe that fecal testing is unsuitable for young people because of the photophobia associated with it. When introducing screening, a balance between sensitivity and specificity, cost, and simplicity should be considered when deciding on the testing method.

### Current Screening Implementation

Many of the communities in this study that screened for *H. pylori* at a young age were using a schooling framework [26, 27, 30, 32, 37–41, 43–61, 63]. Two studies used a pre-enrolment health screening mechanism [34, 35], while others implemented the initiative as part of a health screening during school. Schooling, in which everyone participates, is used to cover all of a generation’s population. In addition, in some areas, *H. pylori* screening for young people was conducted as a part of a screening program for the entire population. Municipalities that are considering implementing *H. pylori* screening should determine what framework they will use and its impact on the population to be targeted, ease of participation in screening, and screening coverage.

The impact of testing and treatment of young asymptomatic patients for *H. pylori* on the prevention of future gastric cancer is not well understood. While there have been efforts to screen young people for *H. pylori* to reduce the risk of future gastric cancer and prevent infection, some reports have suggested that *H. pylori* testing and treatment should not be performed, at least in asymptomatic children, because of concerns regarding the overuse of antibiotics and subsequent side effects. [71]

None of the literature collected for the current study assessed the mid- to long-term impact of screening for *H. pylori* in young people. How screening and treatment of healthy children and adolescents for *H. pylori* will contribute to reducing the risk of future cancer, including long-term adverse events, remains to be studied.

Compared with testing of adults, testing of adolescents and children may require additional considerations. None of the papers in this study used invasive methods such as endoscopy, which is different from testing for adults. There were no reports of major adverse occurrences in any of the studies included in this review. However, special attention should be paid to the occurrence of adverse events as a result of screening, especially in younger generations. The main reason for endoscopy in adults prior to *H. pylori* eradication is to screen for any potential gastric cancer that may have already developed. In contrast, in adolescents, the likelihood of this is low, and thus, the benefits of endoscopy are greatly limited. At the very least, a credible and safe method should be used for *H. pylori* screening in younger populations. For this reason, it is reasonable to use a combination of urine and blood tests, urea breath tests, and stool tests to diagnose and eradicate *H. pylori*.

Finally, none of the studies included in this review compared and discussed the cost of the different types of tests. When administrations initiate screening for disease, its cost-effectiveness needs to be considered. The price of the test itself, the sensitivity and specificity of the test, the time it takes to perform the test, and adherence to the test should be considered when deciding on the screening method. A strategy that combines several tests to avoid the possibility of false-negative results is ideal. [72]

### Limitations

The current study has several limitations. First, the current review was based on studies in which the *H. pylori* test was performed on a population with a thoroughness. This may result in fewer reports than those that have actually been screened. In addition, the results of the present study are not representative of all regions, as report bias may occur. This study includes Japanese literature, and therefore the study may be more relevant in Japan. Stomach cancer is more common in Japan, and more papers have been published on the pros and cons of screening adolescents for *H. pylori*, so we judged that this would provide important insights. A more comprehensive survey is to achieve a thorough overview of *H. pylori* screening in young people worldwide.

## Conclusions

Screening for *H. pylori* in young people was reported from countries around the world. Various methods were used, including urinary antibodies, blood antibodies, stool antigens, and the urea breath test, and less invasive methods should be considered for *H. pylori* screening in young people. Studies observing the effects of *H. pylori* screening on young people in the medium- to long-term were scarce, and as such, further research is required to ascertain these effects. Future study should be conducted in collaboration with the local government, which is the main body of the health care management for young people.

## Supplementary Information

Below is the link to the electronic supplementary material.

Supplementary file1 (DOC 69 KB)

Supplementary file2 (DOCX 15 KB)

Supplementary file3 (DOCX 26 KB)

Supplementary file4 (DOCX 26 KB)

## Data Availability

All data generated or analyzed during this study are included in this published article (and its supplementary information files).
