# Correction to: *Helicobacter pylori* Infection Mass Screening for Children and Adolescents: a Systematic Review of Observational Studies

**DOI:** 10.1007/s12029-021-00636-8

**Published:** 2021-04-21

**Authors:** Hiroaki Saito, Yoshitaka Nishikawa, Yuko Masuzawa, Masaharu Tsubokura, Yasuhiro Mizuno

**Affiliations:** 1 Department of Gastroenterology, Sendai Kousei Hospital, Sendai, Miyagi Japan; 2 Department of Radiation Health Management, Fukushima Medical University School of Medicine, Fukushima, Japan; 3 Department of Health Informatics, Kyoto University School of Public Health, Kyoto, Japan; 4 Chiba Faculty of Nursing, Tokyo Healthcare University, Funabashi, Chiba Japan; 5 Marru-Clinic Yokosuka, Yokosuka, Kanagawa Japan

## Correction to: Journal of Gastrointestinal Cancer https://doi.org/10.1007/s12029-021-00630-0

The article “*Helicobacter pylori* Infection Mass Screening for Children and Adolescents: a Systematic Review of Observational Studies,” written by Hiroaki Saito, Yoshitaka Nishikawa, Yuko Masuzawa, Masaharu Tsubokura, and Yasuhiro Mizuno, was originally published electronically on the publisher’s internet portal on 24 March 2021 without open access. With the author(s)’ decision to opt for Open Choice, the copyright of the article changed on 24 March 2020 to © The Author(s) 2021 and the article is forthwith distributed under a Creative Commons Attribution 4.0 International License, which permits use, sharing, adaptation, distribution, and reproduction in any medium or format, as long as you give appropriate credit to the original author(s) and the source, provide a link to the Creative Commons licence, and indicate if changes were made. The images or other third party material in this article are included in the article’s Creative Commons licence, unless indicated otherwise in a credit line to the material. If material is not included in the article’s Creative Commons licence and your intended use is not permitted by statutory regulation or exceeds the permitted use, you will need to obtain permission directly from the copyright holder. To view a copy of this licence, visit http://creativecommons.org/licenses/by/4.0.

The online version of the original article can be found at https://doi.org/10.1007/s12029-021-00630-0

