# Supplementary material for: Helicobacter pylori Infection Mass Screening for Children and Adolescents: a Systematic Review of Observational Studies
Source: J Gastrointest Cancer. 2021 Mar 24;52(2):489–97. doi: 10.1007/s12029-021-00630-0 (PMC8131279; doi:10.1007/s12029-021-00630-0)
Supplement: Supplementary file 2 — Supplementary file2 (DOCX 15 KB) [file 12029_2021_630_MOESM2_ESM.docx]

**Background**

The mass screening of *Helicobacter pylori (H. pylori)* and eradication for adults has been introduced in areas with high gastric cancer incidence. However, little has been known for the current status of *H. pylori* mass screening for young people.

**Objectives**

We aim to systematically review studies about *Helicobacter. pylori* population-based screening for children and adolescents and clarify the current status of the screening worldwide.

**Methods**

**Criteria for considering studies for this review**

*Types of studies*

We will include available published or unpublished observational studies assessing the H. pylori infection in this review. And conference reports will be also included.

We will exclude review, survey reports for the purpose of infection rate identification, and case reports of less than 10 cases.

*Types of participants*

We will include the healthy populations, including teens, screened for H. pylori (with the town or school as the smallest population unit). Patients with symptoms will be excluded.

*Search methods for identification of studies*

We will conduct a search to identify studies. We will be restricted the language in English or Japanese. We will search the following databases: MEDLINE (via PubMed), EMBASE, Cochrane Library, and ICHUSH using the search terms “H. pylori infection” and “child.

We will be handsearching additional references from The Japanese Journal of Helicobacter Research and The Journal of Japanese Gastroenterological Association.

**Data collection and analysis**

*Selection of studies*

Two authors (HS, YN) independently will assess all potential studies that identified by our search strategy for inclusion in this review. We will resolve any disagreement through discussion or by consulting the third author (YM).

*Data extraction and management*

HS will extract the data by using the standardized form. The items of this form include study design, countries, cities, participants, methods used for testing (e.g. urine, blood, urea breath), and positive rate.

*Assessment of risk of bias in included studies*

Because this review included observational studies using a variety of methods, we will not evaluate using the Risk of Bias tool.

*Data synthesis*

Descriptive summaries of the individual study results will be provided for each outcome in a table that includes the characteristics of each review, by country and by region within Japan.
