# Supplementary material for: Helicobacter pylori Infection Mass Screening for Children and Adolescents: a Systematic Review of Observational Studies
Source: J Gastrointest Cancer. 2021 Mar 24;52(2):489–97. doi: 10.1007/s12029-021-00630-0 (PMC8131279; doi:10.1007/s12029-021-00630-0)
Supplement: Supplementary file 3 — Supplementary file3 (DOCX 26 KB) [file 12029_2021_630_MOESM3_ESM.docx]

Appendix 1.　Search Strategies (December 10, 2018)

【Cochrane Database of Systematic Reviews : Issue 12 of 12, December 2018】

#1 (student*):ti,ab,kw 27042

#2 (child*):ti,ab,kw 115548

#3 (adolescent*):ti,ab,kw 119694

#4 (minor*):ti,ab,kw 16953

#5 (juvenile*):ti,ab,kw 2549

#6 (teenagger*):ti,ab,kw 0

#7 ("young adult*"):ti,ab,kw 66454

#8 (youth*):ti,ab,kw 5100

#9 (school*):ti,ab,kw 24784

#10 MeSH descriptor: [Students] explode all trees 3795

#11 MeSH descriptor: [Child] explode all trees 1408

#12 MeSH descriptor: [Adolescent] explode all trees 97934

#13 MeSH descriptor: [Young Adult] explode all trees 463

#14 MeSH descriptor: [Minors] explode all trees 8

#15 MeSH descriptor: [Schools] explode all trees 2573

#16 MeSH descriptor: [School Health Services] explode all trees 1380

#17 #1 OR #2 OR #3 OR #4 OR #5 OR #6 OR #7 OR #8 OR #9 OR #10 OR #11 OR #12 OR #13 O #14 OR #15 OR #16 264888

#18 ("Helicobacter pylori"):ti,ab,kw 4098

#19 (Helicobacter Infection*):ti,ab,kw 3192

#20 ("H.pylori"):ti,ab,kw 3017

#21 MeSH descriptor: [Helicobacter pylori] explode all trees 1917

#22 MeSH descriptor: [Helicobacter Infections] explode all trees 2017

#23 #18 OR #19 OR #20 OR #21 OR #22 4577

#24 ("Health Examination"):ti,ab,kw 178

#25 (screen):ti,ab,kw 5270

#26 (screening):ti,ab,kw 31709

#27 ("health check up"):ti,ab,kw 44

#28 (assess*):ti,ab,kw 370106

#29 MeSH descriptor: [Mass Screening] explode all trees 3527

#30 #24 OR #25 OR #26 OR #27 OR #28 OR #29 388745

#31 #17 AND #23 AND #30 318

【MEDLINE (via PubMed) (1966 to 10 December 2018)】

#1 Search student*[Text Word] 　　　　　　　　　　　　　　　　　　　　　　　　　281823

#2 Search child*[Text Word] 　　　　　　　　　　　　　　　　　　　　　　　　　2280367

#3 Search adolescent*[Text Word] 　　　　　　　　　　　　　　　　　　　　　1957023

#4 Search minor*[Text Word] 　　　　　　　　　　　　　　　　　　　　　　　　　285154

#5 Search juvenile*[Text Word] 　　　　　　　　　　　　　　　　　　　　　　　　　85806

#6 Search teenager*[Text Word] 　　　　　　　　　　　　　　　　　　　　　　　　　13344

#7 Search "young adult*"[Text Word] 　　　　　　　　　　　　　　　　　　　　　726519

#8 Search youth*[Text Word] 　　　　　　　　　　　　　　　　　　　　　　　　　70584

#9 Search school*[Text Word] 　　　　　　　　　　　　　　　　　　　　　　　　　293061

#10 Search students[MeSH Terms] 　　　　　　　　　　　　　　　　　　　　　113706

#11 Search child[MeSH Terms] 　　　　　　　　　　　　　　　　　　　　　　　　　1800579

#12 Search adolescent[MeSH Terms] 　　　　　　　　　　　　　　　　　　　　　1901050

#13 Search young adult[MeSH Terms] 　　　　　　　　　　　　　　　　　　　　　705647

#14 Search minors[MeSH Terms] 　　　　　　　　　　　　　　　　　　　　　　　　　2480

#15 Search schools[MeSH Terms] 　　　　　　　　　　　　　　　　　　　　　　　　　108117

#16 Search school health services[MeSH Terms] 　　　　　　　　　　　　　　　　　21901

#17 Search (#1 OR #2 OR #3 OR #4 OR #5 OR #6 OR #7 OR #8 OR #9 OR #10 OR #11 OR #12 OR #13 OR #14 OR #15 OR #16) 　　　　　　　　　　　　　　　　　　　　　　　　　　　　　4160381

#18 Search "Helicobacter pylori"[Text Word] 　　　　　　　　　　　　　　　　　41972

#19 Search Helicobacter Infection*[Text Word] 　　　　　　　　　　　　　　　　　28898

#20 Search "H.pylori"[Text Word] 　　　　　　　　　　　　　　　　　　　　　25582

#21 Search Helicobacter pylori[MeSH Terms] 　　　　　　　　　　　　　　　　　33007

#22 Search Helicobacter Infections[MeSH Terms] 　　　　　　　　　　　　　　　　　28795

#23 Search (#18 OR #19 OR #20 OR #21 OR #22) 　　　　　　　　　　　　　　　　　44117

#24 Search "health examination"[Text Word] 　　　　　　　　　　　　　　　　　3841

#25 Search screen[Text Word] 　　　　　　　　　　　　　　　　　　　　　　　　　113721

#26 Search screening[Text Word] 　　　　　　　　　　　　　　　　　　　　　　　　　540091

#27 Search "health check up"[Text Word] 　　　　　　　　　　　　　　　　　　　　　1217

#28 Search assess*[Text Word] 　　　　　　　　　　　　　　　　　　　　　　　　　2904224

#29 Search mass screening[MeSH Terms] 　　　　　　　　　　　　　　　　　　　　　118608

#30 Search (#24 OR #25 OR #26 OR #27 OR #28 OR #29) 　　　　　　　　　　　　　3406283

#31 Search (#17 AND #23 AND #30) 　　　　　　　　　　　　　　　　　　　　　1911

#32 Search (((#17 AND #23 AND #30))) AND ("1945/01/01"[Date - Create] : "2018/12/10"[Date - Create]) 　　　　　　　　　　　　　　　　　　　　　　　　　　　　　　　　　　　　　1908

【EMBASE (1966 to 10 December 2018)】

#1 student*:ab,kw,ti 　　　　　　　　　　　　　　　　　　　　　　　　　　　　　324707

#2 child*:ab,kw,ti 　　　　　　　　　　　　　　　　　　　　　　　　　　　　　1675807

#3 adolescent*:ab,kw,ti 　　　　　　　　　　　　　　　　　　　　　　　　　　　　　300781

#4 minor*:ab,kw,ti 　　　　　　　　　　　　　　　　　　　　　　　　　　　　　349097

#5 juvenile*:ab,kw,ti 　　　　　　　　　　　　　　　　　　　　　　　　　　　　　94023

#6 teenager*:ab,kw,ti 　　　　　　　　　　　　　　　　　　　　　　　　　　　　　18624

#7 'young adult*':ab,kw,ti 　　　　　　　　　　　　　　　　　　　　　　　　　　　　　110423

#8 youth*:ab,kw,ti 　　　　　　　　　　　　　　　　　　　　　　　　　　　　　79884

#9 school*:ab,kw,ti 　　　　　　　　　　　　　　　　　　　　　　　　　　　　　323305

#10 'student'/exp 　　　　　　　　　　　　　　　　　　　　　　　　　　　　　　　　　227101

#11 'juvenile'/exp 　　　　　　　　　　　　　　　　　　　　　　　　　　　　　3482709

#12 'young adult'/exp 　　　　　　　　　　　　　　　　　　　　　　　　　　　　　265296

#13 'minor'/exp 　　　　　　　　　　　　　　　　　　　　　　　　　　　　　　　　　521

#14 'school'/exp 　　　　　　　　　　　　　　　　　　　　　　　　　　　　　　　　　340608

#15 'school health service'/exp 　　　　　　　　　　　　　　　　　　　　　　　　　22242

#16　#1 OR #2 OR #3 OR #4 OR #5 OR #6 OR #7 OR #8 OR #9 OR #10 OR #11 OR #12 OR #13 OR #14 OR #15 　　　　　　　　　　　　　　　　　　　　　　　　　　　　　　　　　4995877

#17 'helicobacter pylori':ab,kw,ti 　　　　　　　　　　　　　　　　　　　　　　　　　50321

#18 'helicobacter infection*':ab,kw,ti 　　　　　　　　　　　　　　　　　　　　　780

#19 'h pylori':ab,kw,ti 　　　　　　　　　　　　　　　　　　　　　　　　　　　　　35883

#20 'helicobacter pylori'/exp 　　　　　　　　　　　　　　　　　　　　　　　　　51521

#21 'helicobacter infection'/exp 　　　　　　　　　　　　　　　　　　　　　　　　　28270

#22 #17 OR #18 OR #19 OR #20 OR #21 　　　　　　　　　　　　　　　　　　　　　68252

#23 'health examination':ab,kw,ti 　　　　　　　　　　　　　　　　　　　　　　　　　5002

#24 screen:ab,kw,ti 　　　　　　　　　　　　　　　　　　　　　　　　　　　　　159761

#25 screening:ab,kw,ti 　　　　　　　　　　　　　　　　　　　　　　　　　　　　　657575

#26 'health check up':ab,kw,ti 　　　　　　　　　　　　　　　　　　　　　　　　　1799

#27 assess*:ab,kw,ti 　　　　　　　　　　　　　　　　　　　　　　　　　　　　　3668897

#28 'mass screening'/exp 　　　　　　　　　　　　　　　　　　　　　　　　　　　　　220159

#29 #23 OR #24 OR #25 OR #26 OR #27 OR #28 　　　　　　　　　　　　　　　　　4354066

#30 #16 AND #22 AND #29 　　　　　　　　　　　　　　　　　　　　　　　　　2147

#31 #16 AND #22 AND #29 AND [1-1-1947]/sd NOT [10-12-2018]/sd 　　　　　　　　　 2143

【ICHUSHI (1970 to 10 December 2018)】

#1 学童/TA 11,528

#2 児童/TA 21,671

#3 生徒/TA 8,030

#4 学生/TA 76,648

#5 中高生/TA 547

#6 高校/TA 11,986

#7 学校/TA 46,259

#8 18歳未満/TA 357

#9 18才未満/TA 3

#10 小児/TA 286,634

#11 青年/TA 10,545

#12 青少年/TA 1,767

#13 未成年/TA 817

#14 若年/TA 56,954

#15 学生/TH 78,043

#16 小児/TH 107,338

#17 青年/TH 2,566

#18 未成年者/TH 352

#19 学校/TH 22,302

#20 学校保健/TH 24,911

#21 #1 or #2 or #3 or #4 or #5 or #6 or #7 or #8 or #9 or #10 or #11 or #12 or #13 or #14 or #15 or #16 or #17 or #18 or #19 or #20 587,597

#22 ピロリ菌/TA 1,767

#23 ヘリコバクター感染/TA 120

#24 ヘリコバクター症/TA 0

#25 h.pylori/TA 9,745

#26 Helicobacter/TA and pylori/TA 12,503

#27 "Helicobacter pylori"/TH 24,913

#28 ヘリコバクター感染症/TH 21,289

#29 #22 or #23 or #24 or #25 or #26 or #27 or #28 28,118

#30 スクリーニング/TA 50,688

#31 ふるいわけテスト/TA 0

#32 検査/TA 508,782

#33 健診/TA 32,425

#34 検診/TA 69,434

#35 終検/TA 140

#36 健康診断/TA 10,234

#37 公費/TA 738

#38 対策事業/TA 497

#39 集団検診/TH 65,734

#40 #30 or #31 or #32 or #33 or #34 or #35 or #36 or #37 or #38 or #39 653,107

#41 #21 and #29 and #40 339

#42 (#41) and (PDAT=//:2018/12/10) 339
