# Supplementary material for: Helicobacter pylori Infection Mass Screening for Children and Adolescents: a Systematic Review of Observational Studies
Source: J Gastrointest Cancer. 2021 Mar 24;52(2):489–97. doi: 10.1007/s12029-021-00630-0 (PMC8131279; doi:10.1007/s12029-021-00630-0)
Supplement: Supplementary file 4 — Supplementary file4 (DOCX 26 KB) [file 12029_2021_630_MOESM4_ESM.docx]

Excluded studies list

1. Akamatsu Y. [Introduction of Screening Test for H. pylori Infection to School Medical Examinations for the Eradication of Gastric Cancer]. *Journal of Gastrointestinal Cancer Screening*. 2012;50(1):88. (in Japanese)
2. Akamatsu Y. [“Gastric cancer screening and H.pylori gastritis” Gastric cancer screening in young and elderly patients Issues in gastric cancer screening and eradication therapy for gastric cancer]. *Clinics in Gastroenterology*. 2015;18(2):168-172. (in Japanese)
3. Antos D, Crone J, Konstantopoulos N, Koletzko S. Evaluation of a novel rapid one-step immunochromatographic assay for detection of monoclonal Helicobacter pylori antigen in stool samples from children. *J Clin Microbiol*. 2005;43(6):2598‐2601. doi:10.1128/JCM.43.6.2598-2601.2005
4. Aoki R, Iga A, Kubo K, Kamamura M, Honda H. [Gastric cancer screening in young patients with H.pylori gastritis. Gastric cancer screening for H.pylori-infected gastritis in young people]. *Journal of Gastrointestinal Cancer Screening*. 2014;52(3):88. (in Japanese)
5. Aoki R, Seii M, Honda H. [Testing for Helicobacter pylori infection in young people]. *Journal of Gastrointestinal Cancer Screening*. 2018;56(3):441. (in Japanese)
6. Asaka M. [How should H. pylori screening and eradication of H. pylori in junior high school for the prevention of gastric cancer? Gastric Cancer Prevention Strategy of Japan 2016]. *J Jpn Pediatr Soc*. 2016;120(2):207. (in Japanese)
7. Banatvala N, Clements L, Abdi Y, Graham JY, Hardie JM, Feldman RA. Migration and Helicobacter pylori seroprevalence: Bangladeshi migrants in the U.K. *J Infect*. 1995;31(2):133‐135. doi:10.1016/s0163-4453(95)92135-4
8. Benson J, Rahim RA, Agrawal R. Newly arrived refugee children with Helicobacter pylori are thinner than their non-infected counterparts. *Aust J Prim Health*. 2017;23(1):92‐96. doi:10.1071/PY15187
9. Blecker U, Hauser B, Lanciers S, Peeters S, Suys B, Vandenplas Y. The prevalence of Helicobacter pylori-positive serology in asymptomatic children. *J Pediatr Gastroenterol Nutr*. 1993;16(3):252‐256. doi:10.1097/00005176-199304000-00005
10. Blecker U, Lanciers S, Hauser B, Vandenplas Y. The prevalence of Helicobacter pylori positivity in a symptom-free population, aged 1 to 40 years. *J Clin Epidemiol*. 1994;47(10):1095‐1098. doi:10.1016/0895-4356(94)90095-7
11. Buckley MJ, O'Shea J, Grace A, et al. A community-based study of the epidemiology of Helicobacter pylori infection and associated asymptomatic gastroduodenal pathology. *Eur J Gastroenterol Hepatol*. 1998;10(5):375‐379. doi:10.1097/00042737-199805000-00004
12. Casswall TH, Nilsson HO, Bergström M, et al. Evaluation of serology, 13C-urea breath test, and polymerase chain reaction of stool samples to detect Helicobacter pylori in Bangladeshi children. *J Pediatr Gastroenterol Nutr*. 1999;28(1):31‐36. doi:10.1097/00005176-199901000-00009
13. Cherian S, Forbes D, Sanfilippo F, Cook A, Burgner D. Helicobacter pylori, helminth infections and growth: a cross-sectional study in a high prevalence population. *Acta Paediatr*. 2009;98(5):860‐864. doi:10.1111/j.1651-2227.2009.01221.x
14. Cherian S, Forbes D, Sanfilippo F, Cook A, Burgner D. The epidemiology of Helicobacter pylori infection in African refugee children resettled in Australia. *Med J Aust*. 2008;189(8):438‐441.
15. Chimonas MA, Baggett HC, Parkinson AJ, Muth PT, Dunaway E, Gessner BD. Asymptomatic Helicobacter pylori infection and iron deficiency are not associated with decreased growth among Alaska Native children aged 7-11 years. *Helicobacter*. 2006;11(3):159‐167. doi:10.1111/j.1523-5378.2006.00395.x
16. Chinda D, Shimoyama K, Fukuda M. [The reality of H. pylori screening and eradication for young people in an area with high gastric cancer mortality]. *Journal of Japanese Society of Gastroenterology*. 2014;111 (special meeting): A254. (in Japanese)
17. Clemens J, Albert MJ, Rao M, et al. Sociodemographic, hygienic and nutritional correlates of Helicobacter pylori infection of young Bangladeshi children. *Pediatr Infect Dis J*. 1996;15(12):1113‐1118. doi:10.1097/00006454-199612000-00012
18. Daugule I, Rumba I, Ejderhamn J. Previous antibacterial treatment due to concomitant infections in preschool children is associated with a lower Helicobacter pylori positivity. *Scand J Infect Dis*. 2005;37(5):326‐329. doi:10.1080/00365540410021063
19. Do MY, Lee YC, Choi CH, et al. [The Changes in Prevalence and the Related Factors of Helicobacter Pylori Infection in Korean Health Check-Up Subjects During 8 Years]. *Korean J Gastroenterol*. 2009;53(2):76‐83. （in Korean）
20. Escobar-Pardo ML, de Godoy AP, Machado RS, Rodrigues D, Fagundes Neto U, Kawakami E. Prevalence of Helicobacter pylori infection and intestinal parasitosis in children of the Xingu Indian Reservation. *J Pediatr (Rio J)*. 2011;87(5):393‐398. doi:10.2223/JPED.2118
21. Falsafi T, Valizadeh N, Sepehr S, Najafi M. Application of a stool antigen test to evaluate the incidence of Helicobacter pylori infection in children and adolescents from Tehran, Iran. *Clin Diagn Lab Immunol*. 2005;12(9):1094‐1097. doi:10.1128/CDLI.12.9.1094-1097.2005
22. Ghasemi-Kebria F, Ghaemi E, Azadfar S, Roshandel G. Epidemiology of Helicobacter pylori infection among Iranian children. *Arab J Gastroenterol*. 2013;14(4):169‐172. doi:10.1016/j.ajg.2013.11.002
23. Hara H, Ito M, Hamada F, Hosoki H, Yamane T. [Helicobacter pylori infection in children]. *Journal of Kochi Medical Association*. 1999;4(1):51-54. (in Japanese)
24. Hollander W.J., Den and I.L., Holster , et al. Risk factors for helicobacter pylori acquisition in a multi-ethnic urban population. *Gastroenterology*. 2014;146 (5): S-401.
25. Ikuse K. [What should be done for H. pylori screening and eradication for prevention of gastric cancer? Diagnosis of h.pylori infection method and pitfall in sanitization decisions]. *J Jpn Pediatr Soc*. 2016;120(2):208. (in Japanese)
26. J. Gastroenterol. Hepatol. Relationship of intestinal parasites, H. pylori infection with anemia or iron status among school age children in rural Bangladesh. J. *Gastroenterol. Hepatol*. 2013;2 (9): 769-773.
27. Jafar S, Jalil A, Soheila N, Sirous S. Prevalence of helicobacter pylori infection in children, a population-based cross-sectional study in west iran. *Iran J Pediatr*. 2013;23(1):13‐18.
28. Jafri W, Yakoob J, Abid S, Siddiqui S, Awan S, Nizami SQ. Helicobacter pylori infection in children: population-based age-specific prevalence and risk factors in a developing country. *Acta Paediatr*. 2010;99(2):279‐282. doi:10.1111/j.1651-2227.2009.01542.x
29. 29. Jafri W, Yakoob J, Abid S, Siddiqui S, Awan S, Nizami SQ. H. pylori infection in children: Population-based age-specific prevalence and risk factors in a developing country. *Helicobacter.* 2009;14(4):349.
30. Kakiuchi T, Endo H, Matsuo S. [H.pylori screening of all junior high school students in Saga Prefecture]. *Journal of Japanese Society of Gastroenterology*. 2017;114(special meeting):A304. (in Japanese)
31. Kakiuchi T, Endo H, Yoshihara D, et al. [Overview of the "Project to Promote Gastric Cancer Control Measures for the Future" for H. pylori screening for third-year junior high school students in Saga Prefecture]. *J Jpn Pediatr Soc*. 2016;120(12):1829. (in Japanese)
32. Kienesberger S, Perez-Perez GI, Olivares AZ, et al. When is Helicobacter pylori acquired in populations in developing countries? A birth-cohort study in Bangladeshi children. *Gut Microbes*. 2018;9(3):252‐263. doi:10.1080/19490976.2017.1421887
33. Koletzko S, Feydt-Schmidt A. Infants differ from teenagers: use of non-invasive tests for detection of Helicobacter pylori infection in children. *Eur J Gastroenterol Hepatol*. 2001;13(9):1047‐1052. doi:10.1097/00042737-200109000-00008
34. Kotilea K, Mekhael J, Salame A, et al. Eradication rate of Helicobacter Pylori infection is directly influenced by adherence to therapy in children. *Helicobacter*. 2017;22(4):10.1111/hel.12383. doi:10.1111/hel.12383
35. Kukushkina IA, Korotkova OA, Loginov IA, et al. Application of electrochemical breath test for detection of Helicobacter pylori in screening of Moscow students. *Bull Exp Biol Med*. 2012;152(3):325‐328. doi:10.1007/s10517-012-1519-9
36. Kurahara, K. [Introduction of H. pylori testing to school examinations for the eradication of gastric cancer]. *Journal of the Okinawa Medical Association*. 2016;52(3):358. (in Japanese)
37. Lim SH, Kim N, Kwon JW, et al. Trends in the seroprevalence of Helicobacter pylori infection and its putative eradication rate over 18 years in Korea: A cross-sectional nationwide multicenter study. *PLoS One*. 2018;13(10):e0204762. Published 2018 Oct 17. doi:10.1371/journal.pone.0204762
38. Lorenzo I, Fernández-de-Larrea N, Michel A, et al. Helicobacter pylori seroprevalence in Spain: influence of adult and childhood sociodemographic factors. *Eur J Cancer Prev*. 2019;28(4):294‐303. doi:10.1097/CEJ.0000000000000483
39. Mabe K, Kikuchi S, Okuda M, Takamasa M, Kato M, Asaka M. Diagnostic accuracy of urine Helicobacter pylori antibody test in junior and senior high school students in Japan. *Helicobacter*. 2017;22(1):10.1111/hel.12329. doi:10.1111/hel.12329
40. Mabe K, Kikuchi S, Okuda M, Takamasa M, Kato M, Asaka M. Diagnostic accuracy of urine Helicobacter pylori antibody test in junior and senior high school students in Japan. *Helicobacter*. 2017;22(1):10.1111/hel.12329. doi:10.1111/hel.12329
41. Malaty HM, Graham DY, Wattigney WA, Srinivasan SR, Osato M, Berenson GS. Natural history of Helicobacter pylori infection in childhood: 12-year follow-up cohort study in a biracial community. *Clin Infect Dis*. 1999;28(2):279‐282. doi:10.1086/515105
42. Malaty HM, Kumagai T, Tanaka E, et al. Evidence from a nine-year birth cohort study in Japan of transmission pathways of Helicobacter pylori infection. *J Clin Microbiol*. 2000;38(5):1971‐1973.
43. Merino D, Galván M, Fabre A, Balbachán S, Miranda O, Gorodner J, Alonso J. Helicobacter pylori infection in children from Northeast Argentina: Seroprevalence and its relation with nutritional status and socio-sanitary conditions. Enfermedades Emergentes. 2002;4(1):24‐29.
44. Nabwera HM, Nguyen-Van-Tam JS, Logan RF, Logan RP. Prevalence of Helicobacter pylori infection in Kenyan schoolchildren aged 3-15 years and risk factors for infection. *Eur J Gastroenterol Hepatol*. 2000;12(5):483‐487. doi:10.1097/00042737-200012050-00002
45. Nakayama Y, Ikegami M, Taki Y, Kubota S, Kumagai T. [Four cases of h.pylori infection with abnormal blood tests in school screening]. *J Jpn Pediatr Soc*. 2003;107(10):1405. (in Japanese)
46. Namkin K, Zardast M, Basirinejad F. Saccharomyces Boulardii in Helicobacter Pylori Eradication in Children: A Randomized Trial From Iran. *Iran J Pediatr*. 2016;26(1):e3768. doi:10.5812/ijp.3768
47. Okuda M. [Let's give children a future without stomach cancer]. *Journal of Gastrointestinal Cancer Screening*. 2017;55(6):1136. (in Japanese)
48. Ono K, Handa K. [A case study of high negative results in a health screening system for second-year junior high school students using the Helicobacter pylori antibody titer test]. *Journal of Japanese Society of Gastroenterology*. 2017;114(special meeting):A715. (in Japanese)
49. Ozen A, Ertem D, Pehlivanoglu E. Natural history and symptomatology of Helicobacter pylori in childhood and factors determining the epidemiology of infection. *J Pediatr Gastroenterol Nutr*. 2006;42(4):398‐404. doi:10.1097/01.mpg.0000215307.48169.7b
50. Pérez-Pérez GI, Sack RB, Reid R, Santosham M, Croll J, Blaser MJ. Transient and persistent Helicobacter pylori colonization in Native American children. *J Clin Microbiol*. 2003;41(6):2401‐2407. doi:10.1128/jcm.41.6.2401-2407.2003
51. Rowland M, Daly L, Vaughan M, Higgins A, Bourke B, Drumm B. Age-specific incidence of Helicobacter pylori. *Gastroenterology*. 2006;130(1):65‐211. doi:10.1053/j.gastro.2005.11.004
52. Sasaki M, Konno M, Sugawara M, Yokota S. [Clinical examination of childhood serum pepsinogen levels: Comparison with normal and H. pylori-positive children in different age groups]. *HOKKAIDONOUSONIKAISHI*. 2017;49:73-75. (in Japanese)
53. Tkachenko MA, Zhannat NZ, Erman LV, et al. Dramatic changes in the prevalence of Helicobacter pylori infection during childhood: a 10-year follow-up study in Russia. *J Pediatr Gastroenterol Nutr*. 2007;45(4):428‐432. doi:10.1097/MPG.0b013e318064589f
54. Toscano EP, Madeira FF, Dutra-Rulli MP, et al. Epidemiological and Clinical-Pathological Aspects of *Helicobacter pylori* Infection in Brazilian Children and Adults [published correction appears in Gastroenterol Res Pract. 2019 May 2;2019:5632935]. *Gastroenterol Res Pract*. 2018;2018:8454125. Published 2018 Sep 4. doi:10.1155/2018/8454125
55. Tsutsumi K, Kusano C, Suzuki S, Gotoda T, Murakami K. Diagnostic Accuracy of Latex Agglutination Turbidimetric Immunoassay in Screening Adolescents for Helicobacter pylori Infection in Japan. *Digestion*. 2018;98(2):75‐80. doi:10.1159/000487184
56. Yamawaki, H. [School health page: Testing of H. pylori bacteria in junior high school students in Mito City]. *The Journal of the Ibaraki Medical Association*. 2017(767):33. (in Japanese)
57. Yilmaz E, Doğan Y, Gürgöze MK, Unal S. Seroprevalence of Helicobacter pylori infection among children and their parents in eastern Turkey. *J Paediatr Child Health*. 2002;38(2):183‐186.
58. Yoshitoshi T., Iwama T., Tsuji Y., et al. [Current status of pediatric Helicobacter pylori infection treatment]. *J Jpn Pediatr Soc*. 2017;121(11):1835-1840. (in Japanese)
